# Description and complete genome sequences of *Bradyrhizobium symbiodeficiens* sp. nov., a non-symbiotic bacterium associated with legumes native to Canada

**DOI:** 10.1099/ijsem.0.003772

**Published:** 2020-02-03

**Authors:** Eden S. P. Bromfield, Sylvie Cloutier, Hai D. T. Nguyen

**Affiliations:** ^1^​ Ottawa Research and Development Centre, Agriculture and Agri-Food Canada, 960 Carling Avenue, Ottawa, Ontario K1A OC6, Canada

**Keywords:** *Bradyrhizobium symbiodeficiens*, complete genome sequence, non-symbiotic

## Abstract

Four bacterial strains isolated from root nodules of soybean plants that had been inoculated with root-zone soil of either *Amphicarpaea bracteata* (Hog Peanut) or *Desmodium canadense* (Showy Tick Trefoil) growing in Canada, were previously characterized and placed in a novel lineage within the genus *Bradyrhizobium *. The taxonomic status of the novel strains was verified by genomic and phenotypic analyses. Phylogenetic analyses of individual and concatenated housekeeping gene sequences (*atp* *D*, *gln* *II*, *rec* *A*, *gyr* *B* and *rpo* *B*) placed all novel strains in a highly supported lineage distinct from named *Bradyrhizobium* species. Data for sequence similarities of concatenated housekeeping genes of novel strains relative to type strains of named species were consistent with the phylogenetic data. Average nucleotide identity values of genome sequences (84.5–93.7 %) were below the threshold value of 95–96 % for bacterial species circumscription. Close relatives to the novel strains are *Bradyrhizobium amphicarpaeae *, *Bradyrhizobium ottawaense* and *Bradyrhizobium shewense *. The complete genomes of strains 85S1MB^T^ and 65S1MB consist of single chromosomes of size 7.04 and 7.13 Mbp, respectively. The genomes of both strains have a G+C content of 64.3 mol%. These strains lack a symbiosis island as well as key nodulation, nitrogen-fixation and photosystem genes. Data from various phenotypic tests including growth characteristics and carbon source utilization supported the sequence-based analyses. Based on the data presented here, the four strains represent a novel species for which the name *B* *radyrhizobium* *symbiodeficiens* sp. nov., is proposed, with 85S1MB^T^ (=LMG 29937^T^=HAMBI 3684^T^) as the type strain.

Legumes native to east North America such as *Amphicarpaea bracteata* (Hog Peanut) and *Desmodium canadense* (Showy tick trefoil) form symbiotic associations with diverse species of soil bacteria belonging to the genus *Bradyrhizobium* [1–3]. In a previous study [3] bacteria were isolated from root nodules of soybean plants that had been inoculated with root-zone soils of several legume species native to eastern Canada. Bacteria were characterized on the basis of multilocus sequence analysis (MLSA) of five protein encoding housekeeping genes and multiple novel lineages in the genus *Bradyrhizobium* were identified. One of these novel lineages, consisted of four strains that lacked key nodulation (*nod*) and nitrogen-fixation (*nif*) genes and did not elicit root nodules on any of the legume hosts tested. In this work we used genomic and phenotypic analyses to further characterize strains of this novel lineage and based on the results, the species, *Bradyrhizobium symbiodeficiens* sp. nov., is proposed.

## Habitat and isolation

Four novel strains were isolated from root nodules of soybean plants that had been inoculated with suspensions of root zone soil from either *A. bracteata* (strains 65S1MB, 85S1MB^T^ and 101S1MB) or *D. canadense* (strain 141S2) plants growing in natural woodland sites in Canada [3]. The type strain was deposited in the BCCM/LMG Bacteria Collection, University of Gent, Belgium (LMG no. 29937) and in the hambi Microbial Culture Collection, University of Helsinki, Finland (hambi no. 3648).

## Phylogenetic characterization

Sequences of the 16S rRNA, *atp* *D*, *gln* *II*, *gyr* *B*, *rec* *A* and *rpo* *B* housekeeping genes were used for phylogenetic analyses. GenBank accession numbers of nucleotide sequences are shown in Table S1. Sequences were aligned as described previously [2, 4]. Best fit substitution models were selected using algorithms implemented in JModelTest version 2.1.10 [5]. Bayesian phylogenetic analyses were carried out using MrBayes version 3.2.1 with default priors [6] as described in a previous study [4]. Maximum-likelihood (ML) phylogenetic analyses [7] were carried out using 1000 non-parametric bootstrap replications to assess support [2]. The topologies of the Bayesian and ML phylogenetic trees were similar and therefore only Bayesian trees are shown in this work.

In order to reconstruct a 16S rRNA gene tree of type strains of all named *Bradyrhizobium* species (Table S1, available in the online version of this article), it was necessary to trim the aligned length of sequences to 1238 bp. The Bayesian tree of 16S rRNA gene sequences (Fig. S1) showed that type strains of all *Bradyrhizobium* species were placed in two superclades: one represented by *Bradyrhizobium japonicum* and the other by *Bradyrhizobium elkani*. All four novel strains had identical 16S rRNA gene sequences and were placed in the superclade represented by *B. japonicum *. It should be noted, however, that the 16S rRNA gene is highly conserved and its usefulness as a taxonomic marker for species differentiation in the genus *Bradyrhizobium* is limited [8]. Sequence similarities for the 16S rRNA gene of novel strains versus type strains of *Bradyrhizobium* species, computed using software implemented in EzBioCloud [9], are in accord with the phylogenetic data (Table 1).

**Table 1.** Sequence similarities (%) for two and five concatenated housekeeping genes and 16S rRNA genes of *Bradyrhizobium* *symbiodeficiens* sp. nov.*,* 85S1MB^T^ versus reference taxa NA, Sequence not available in public databases.

Sequence similarity (%) to *Bradyrhizobium symbiodeficiens* 85S1MB^T^ (% coverage if not 100*)							
Strain	Five genes† (2679 bp)	Two genes† (854 bp)	16S rRNA gene (1422 bp)	Strain	Five genes† (2679 bp)	Two genes† (854 bp)	16S rRNA gene (1422 bp)
***Bradyrhizobium symbiodeficiens*** **65** **S1MB**	99.2	99.0	100.0	* Bradyrhizobium* *japonicum * USDA6^T^	94.2	94.4	99.8
***Bradyrhizobium symbiodeficiens*** **101S1MB**	99.3	98.7	100.0	* Bradyrhizobium* *jicamae * PAC68^T^	89.7	89.8	97.9
*** B* *radyrhizobium*** ***symbiodeficiens*** **141S2**	99.4	98.1	100.0	* Bradyrhizobium* *kavangense * 14-3^T^	93.2	92.6	94.9 (95)
* Bradyrhizobium algeriense * RST89^T^	89.2	89.8	98.1‡(92)	* Bradyrhizobium* *lablabi * CCBAU23086^T^	89.9	90.4	97.6 (96)
* Bradyrhizobium* *americanum * CMVU44^T^	na	93.4	99.4	* Bradyrhizobium* *liaoningense * LMG18230^T^	94.4	94.3	99.7
* Bradyrhizobium* *amphicarpaeae * 39S1MB^T^	97.0	96.8	99.6	* Bradyrhizobium* *lupini * USDA3051^T^	na	93.1	99.7
* Bradyrhizobium* *arachidis * LMG26795^T^	94.4	94.4	99.0	* Bradyrhizobium* *macuxiense* BR10303^T^	90.4	89.7	97.4‡(91)
* Bradyrhizobium* *betae * LMG21987^T^	94.6	95.1	99.2	* Bradyrhizobium* *manausense * HAMBI 3596^T^	93.0	92.3	98.9
* Bradyrhizobium* *brasilense* UFLA03-321^T^	90.9	89.7	97.5†	* Bradyrhizobium* *mercantei * SEMIA 6399^T^	90.4	89.8	97.8
* Bradyrhizobium* *cajani * AMBPC1010^T^	na	94.0	99.2	* Bradyrhizobium* *namibiense * 5-10^T^	88.8	88.6	97.8
* Bradyrhizobium* *canariense * BTA-1^T^	93.1	93.8	99.6	* Bradyrhizobium* *neotropicale * HAMBI 3599^T^	93.1	92.6	97.4
* Bradyrhizobium* *centrolobii* BR10245^T^	93.0	92.6	96.6‡(93)	* Bradyrhizobium* *nitroreducens* TSA1^T^	94.6	93.0	99.6‡
* Bradyrhizobium* *centrosemae * A9^T^	na	92.3	99.4	* Bradyrhizobium* *oligotrophicum * LMG10732^T^	89.4	87.9	98.6
* Bradyrhizobium* *cytisi * CTAW11^T^	93.3	92.4	99.2	* Bradyrhizobium* *ottawaense * OO99^T^	95.2	94.6	99.9
* Bradyrhizobium* *daqingense * CCBAU15774^T^	93.4	93.2	99.4	* Bradyrhizobium* *pachyrhizi * PAC48^T^	90.6	89.5	97.5
* Bradyrhizobium* *denitrificans * LMG8443^T^	89.8	87.9	98.4	* Bradyrhizobium* *paxllaeri * LMTR21^T^	89.5	90.2	97.8
* Bradyrhizobium* *diazoefficiens * USDA110^T^	94.9	95.1	99.2	* Bradyrhizobium* *retamae * Ro19^T^	88.4	89.1	98.1
* Bradyrhizobium* *elkanii * USDA76^T^	91.1	89.9	97.5	* Bradyrhizobium* *rifense * CTAW71^T^	93.7	92.4	99.3
* Bradyrhizobium* *embrapense * CNPSo 2833^T^	90.6	90.4	97.8	* Bradyrhizobium* *ripae * WR4^T^	na	89.6	97.5‡
* Bradyrhizobium* *erythrophlei * CCBAU 53325^T^	na	89.5	98.1‡ (94)	* Bradyrhizobium* *sacchari* BR10280^T^	93.7	93.4	98.8‡(96)
* Bradyrhizobium* *ferriligni * CCBAU 51502^T^	na	89.7	95.6 (94)	* Bradyrhizobium* *shewense * ERR11^T^	95.7	94.6	99.8
* Bradyrhizobium* *forestalis* NPA54B^T^	93.3	93.4	99.7‡	* Bradyrhizobium* *stylosanthis * BR446^T^	94.3	94.0	98.8 (93)
* Bradyrhizobium* *ganzhouense * CCBAU101088^T^	na	93.4	99.1 (97)	* Bradyrhizobium* *subterraneum * 58 2-1^T^	na	93.0	99.7 (95)
* Bradyrhizobium* *guangdongense * CCBAU51649^T^	na	91.2	99.1 (90)	* Bradyrhizobium tropiciagri * CNPSo1112^T^	91.2	90.3	97.5
* Bradyrhizobium* *guangxiense * CCBAU53363T	na	92.9	99.4 (89)	* Bradyrhizobium* *valentinum * LMG27619^T^	88.8	88.2	97.7
* Bradyrhizobium* *huanghuaihaiense* CCBAU23303^T^	94.3	94.4	99.1	* Bradyrhizobium* *vignae * 7-2^T^	93.1	93.3	98.9 (92)
* Bradyrhizobium* *icense * HAMBI 3584^T^	88.8	89.6	97.8	* Bradyrhizobium* *viridifuturi * SEMIA690^T^	91.2	89.8	97.8
* Bradyrhizobium* *ingae * HAMBI3600^T^	na	93.1	98.5 (96)	* Bradyrhizobium* *yuanmingense * CCBAU10071^T^	93.7	93.6	99.6
* Bradyrhizobium* *iriomotense * EK05^T^	92.4	92.5	98.6				

*Short sequences.

**Fig. 1.**
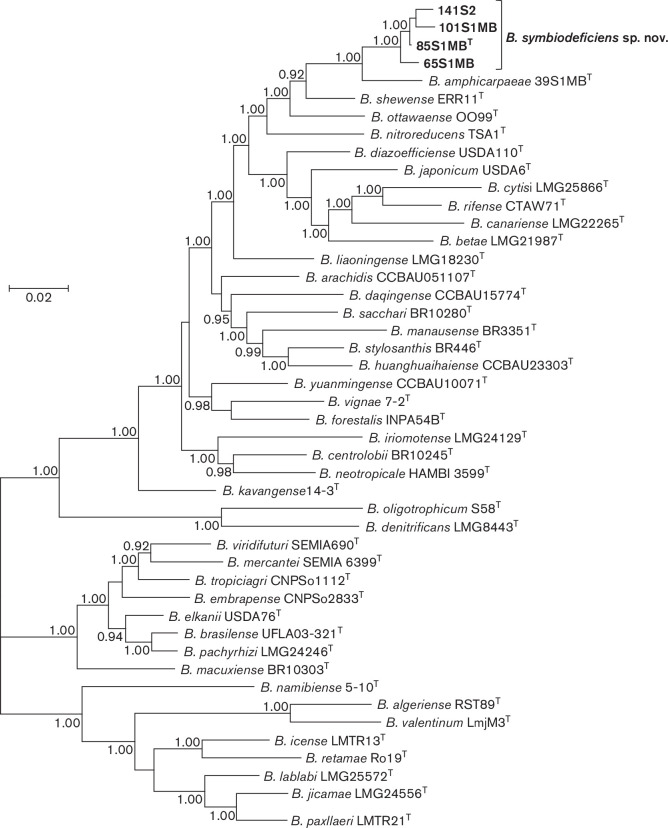
Bayesian phylogenetic tree (GTR+G+I substitution model) of *at* *pD-glnII-recA-gyrB-rpoB* concatenated housekeeping gene sequences (2679 bp) for *Bradyrhizobium symbiodeficiens* sp. nov. and reference taxa of the genus *Bradyrhizobium *. Posterior probabilities ≥90 % are shown. Bar, expected substitutions per site.

†Two concatenated genes: *rec*A and *gln*II (Fig. S7); five concatenated genes: *atp*D, *gln*II, *gyr*B, *rec*A and *rpo*B (Fig. 1).

‡16S rRNA gene similarities calculated using sequences from GenBank as reference sequences not available inEzBioCloud database [8].

Phylogenetic analysis based on MLSA of at least five protein encoding housekeeping genes is a reliable and powerful method for the delineation of species within the genus *Bradyrhizobium* [2, 10, 11]. Corroborating our previous work [3], the Bayesian phylogenetic tree of five concatenated housekeeping gene sequences (Fig. 1) as well as trees for individual gene sequences (Figs. S2–S6), placed the four novel strains in a highly supported lineage (100 % posterior probability) distinct from named *Bradyrhizobium* species. The closest relatives of novel strains are *B. amphicarpaeae* 39S1MB^T^, *B. ottawaense* OO99^T^ and *B. shewense* ERR11^T^.

As one or more protein encoding housekeeping gene sequences for type strains of several *Bradyrhizobium* species are not available in public databases, we reconstructed a supplementary phylogenetic tree using the two gene sequences (*rec* *A* and *gln* *II*) that are available for all named species. In order to include type strains of all these species in the analysis, it was necessary to trim aligned sequence lengths to 375 and 479 bp for the *rec* *A* and *gln* *II* genes, respectively. The Bayesian tree of *rec* *A*-*gln* *II* concatenated gene sequences confirmed the placement of novel strains in a lineage that is distinct from named species of the genus *Bradyrhizobium* (Fig. S7).

It is noteworthy that recent phylogenomic studies [12, 13] using multiple protein encoding gene sequences from bacterial genomes available in public databases also place strain 85S1MB^T^ in a novel lineage within the genus *Bradyrhizobium *.

Sequence similarities for pair-wise comparisons of novel strain 85S1MB^T^ with reference taxa for two and five concatenated housekeeping gene sequences were calculated using the method of Stothard [14]. Table 1 shows that relative to 85S1MB^T^, novel strains 65S1MB, 101S1MB and 141S2 had sequence similarities for the five concatenated housekeeping genes that were >99 % whereas in comparisons with type strains of named species similarity values were at or below the 97 % cut-off value proposed for species delineation in the genus *Bradyrhizobium* [11]; similar results were obtained for comparisons using two concatenated housekeeping genes.

Each of the four novel strains are genetically distinct and were assigned to a unique multilocus sequence genotype based on MLSA of concatenated housekeeping genes (Fig. 1).

Genetic differences between novel strains were further verified by generating random amplified polymorphic DNA (RAPD) fingerprints using four random primers (P1, P2, P3 and P5) and the amplification methods described previously [4]. An example of the fingerprint profiles generated by one of the primers (P5) is shown in Fig. S8.

A dendrogram based on the combined character matrix of fingerprint profiles generated by the four primers was reconstructed using UPGMA and the Dice coefficient implemented in GelCompare II software version 5.10 (Applied Maths). The novel strains were readily distinguished and were placed in a cluster distinct from reference taxa (Fig. S9).

## Genomic characterization

For further characterization, we sequenced the complete genomes of novel strains 85S1MB^T^ and 65S1MB. Sequencing was done at Genome Quebec Innovation Centre, Montreal, Canada, using the Pacific Biosciences (PacBio) RS II single-molecule real-time (smrt) platform [15] as described previously [16]. One smrt cell was used for strain 85S1MB^T^ whereas two smrt cells were used for strain 65S1MB.

Average nucleotide identity (ANI) of genomic sequences is recommended as a replacement for outdated DNA–DNA hybridization methods for bacterial species circumscription [8, 17–19]. We estimated ANI values for the complete genome sequences of 85S1MB^T^ and 65S1MB in pair-wise comparisons with genome sequences of type strains of named *Bradyrhizobium* species obtained from public databases using the MUMmer (ANIm) algorithm implemented in J-species Web Server version 3.0.20 [20]. Table 2 shows that relative to novel strains 85S1MB^T^ and 65S1MB, the ANI values of named species varied between 84.5 % (* B. icense *) and 93.7 % (* B. amphicarpaeae *), which is below the threshold value of 95–96 % for bacterial species circumscription [8, 19, 21]. In contrast, the ANI value of 98.6 % for the comparison of novel strains 85S1MB^T^ versus 65S1MB, is consistent with these strains belonging to the same species.

**Table 2.** Average nucleotide identity (ANI) values for pair-wise comparisons of the complete genome sequences of *Bradyrhizobium* *symbiodeficiens* sp. nov. strains 85S1MB^T^ and 65S1MB versus named *Bradyrhizobium* species

Reference strain (accession no.)	ANI (%)		Reference Strain (accession no.)	ANI (%)	
	85S1MB^T^	65S1MB		85S1MB^T^	65S1MB
***Bradyrhizobium symbiodeficiens* 85S1MB^T^ (CP029427**)	–	**98.6**	* Bradyrhizobium elkanii* USDA76^T^ (ARAG01000000)	85.4	85.4
***Bradyrhizobium symbiodeficiens* 65S1MB (CP041090**)	**98.6**	–	*Bradyrhizobium brasilense* UFLA03-321^T^ (MPVQ00000000)	85.3	85.3
* Bradyrhizobium amphicarpaeae* 39S1MB^T^ (CP029426)	93.7	93.7	* Bradyrhizobium pachyrhizi* LMG24246^T^ (LFIQ00000000)	85.3	85.3
* Bradyrhizobium ottawaense* OO99^T^ (CP029425)	92.0	91.9	* Bradyrhizobium embrapense* CNPSo2833^T^ (LFIP00000000)	85.2	85.3
* Bradyrhizobium shewense* ERR11^T^ (FMAI00000000)	91.9	91.9	*Bradyrizobium mercantei* SEMIA 6399^T^ (MKFI00000000)	85.2	85.3
*Bradyrhizobium nitroreducens* TSA1^T^ (LFJC00000000)	90.4	90.3	* Bradyrhizobium tropiciagri* CNPSo1112^T^ (LFLZ00000000)	85.2	85.3
*Bradyrhizobium forestalis* INPA54B^T^ (PGVG00000000)	89.3	89.3	*Bradyrizobium viridifuturi* SEMIA690^T^ (LGTB00000000)	85.2	85.2
* Bradyrhizobium diazoefficiens* USDA110^T^ (BA000040)	89.2	89.2	*Bradyrhizobium macuxiense* BR10303^T^ (LNCU00000000)	85.1	85.1
*Bradyrizobium japonicum* USDA6^T^ (AP012206)	89.0	89.0	*Bradyrizobium oligotrophicum* LMG10732^T^ (AP012603)	84.8	84.8
* Bradyrhizobium stylosanthis* BR446^T^ (LVEM00000000)	89.0	89.0	* Bradyrhizobium lablabi* CCBAU23086^T^ (LLYB00000000)	84.7	84.7
* Bradyrhizobium arachidis* CCBAU051107^T^ (FPBQ00000000)	88.9	88.8	* Bradyrhizobium jicamae* PAC68^T^ (LLXZ00000000)	84.7	84.7
* Bradyrhizobium vignae* 7-2^T^ (RDQF00000000)	88.9	88.9	*Bradyrizobium paxllaeri* LMTR21^T^ (MAXB00000000)	84.7	84.7
* Bradyrhizobium yuanmingense* CCBAU10071^T^ (FMAE00000000)	88.8	88.8	* Bradyrhizobium algeriense* RST89^T^ (PYCM00000000)	84.6	84.6
*Bradyrhizobium sacchari* BR10280^T^ (LWIG00000000)	88.7	88.7	* Bradyrhizobium valentinum* LmjM3^T^ (LLXX00000000)	84.5	84.5
* Bradyrhizobium manausense* HAMBI 3596^T^ (LJYG00000000)	87.7	87.7	* Bradyrhizobium retamae* Ro19^T^ (LLYA00000000)	84.5	84.5
* Bradyrhizobium neotropicale* HAMBI 3599^T^ (LSEF00000000)	87.6	87.5	* Bradyrhizobium icense* HAMBI 3584^T^ (CP016428)	84.5	84.5
*Bradyrhizobium centrolobii* BR10245^T^ (LUUB00000000)	87.6	87.6			

The complete genomes of novel strains 85S1MB^T^ and 65S1MB consist, respectively, of single circular chromosomes of 7039503 bp and 7133277 bp, which are similar in size to their close relative, *B. amphicarpaeae* 39S1MB^T^ (7044517 bp) but smaller than the chromosomes of *B. ottawaense* OO99^T^ (8606328 bp) and *B. shewense* ERR11^T^ (9163226 bp) (Table 3). Novel strains 85S1MB^T^ and 65S1MB do not possess plasmids. The G+C content of strains 85S1MB^T^ and 65S1MB is 64.3 %, which is within the range for members of the genus *Bradyrhizobium *. Estimated genome coverage for strain 85S1MB^T^ was 90-fold with 102 625 polymerase reads and an average read length of 9102 bp; for strain 65S1MB coverage was 111-fold with 91 331 polymerase reads and an average read length of 12883 bp. Totals of 6682 genes, 6625 coding sequences, 50 tRNAs, and three rRNA operons (5, 16 and 23S) were found for strain 85S1MB^T^ whereas 6795 genes, 6507 coding sequences, 51 tRNAs, and three rRNA operons (5, 16 and 23S) were found for strain 65S1MB using the ncbi Prokaryotic Genome Annotation Pipeline version 4 (PGAP-4) [22, 23]. Analyses carried out with the patric version 3.5.26 platform [24] indicated that the most abundant genes for strains 85S1MB^T^ and 65S1MB, respectively, were those involved in metabolism (900 and 926 genes), energy (297 and 300 genes), protein processing (234 and 233 genes), membrane transport (215 and 235 genes) and cellular processes (181 and 179 genes). Also found were genes implicated in stress response, defense and virulence (155 and 157 genes), motility and chemotaxis (82 and 82 genes), regulation and cell signalling (23 and 23 genes), and resistance to antibiotics and toxic compounds (57 and 58 genes).

**Table 3.** Characteristics of genome sequences of *Bradyrhizobium* *symbiodeficiens* sp. nov., strains 85S1MB^T^ (accession no. CP029427) and 65S1MB (accession no. CP041090), and close relatives *Bradyrhizobium* *amphicarpaeae* 39S1MB^T^ (accession no. CP029426), *Bradyrhizobium* *ottawaense* OO99^T^ (accession no. CP029425) and *Bradyrhizobium* *shewense* ERR11^T^ (accession no. FMAI01000000) Unless otherwise stated, data are from the ncbi assembly databases. na, Data not available.

Characteristic	Strain				
	85S1MB^T^	65S1MB	39S1MB^T^	OO99^T^	ERR11^T^
Genome assembly quality	Complete	Complete	Complete	Complete	Draft
Genome size (bp)	7039503	7133277	7044517	8606328	9163226
Genes (total)	6682	6795	6635	8238	8634*
CDS (total)	6625	6737	6579	8180	9479†
CDS (coding)	6414	6507	6441	7700	8548*
Genes (RNA)	57	58	56	58	86*
rRNAs	3	3	3	3	3†
tRNAs	50	51	49	51	57†
Pseudo genes (total)	211	230	138	480	na
Repeat regions†	30	34	48	177	231
DNA G+C content†	64.27	64.34	64.66	63.83	63.22
Plasmids	No	No	No	No	na
Photosynthetic gene cluster	No	No	Yes	No	No
Symbiosis island	No	No	No	Yes	Yes

*Data from Aserse *et al*. [19].

†Data from the patric Bioinformatics Database [22].

A total of five (strain 85S1MB^T^) and seven (strain 65S1MB) genomic islands were predicted based on the Island Path-DIMOB software implemented in the IslandViewer 4 platform [25], but unlike type strains of several symbiotic species such as *B. ottawaense* and *B. diazoefficiens *, the genomes of novel strains do not contain a symbiosis island, key nodulation (*nodDYABCSUIJ*) or nitrogen-fixing (*nifDKEN*, *nifH*, *nifA* and *fixABCX*) genes. Within the genus *Bradyrhizobium *, *nod* and *nif* genes have also been reported to be absent from diverse *Bradyrhizobium* spp. resident in North American forest soils [26] and these genes have not been detected in *B. betae* PL7HG1^T^ isolated from a tumour on the roots of sugar beet [27].

Genes for Type I and II/IV secretion systems were found in the genomes of strains 85S1MB^T^ and 65S1MB, but not genes for Type III secretion systems. Unlike the close relative, *B. amphicarpaeae* 39S1MB^T^ [28], strains 85S1MB^T^ and 65S1MB do not possess photosystem genes (Table 3). As such, the novel strains should prove useful for studies on the evolution of symbiosis, nitrogen-fixation and photosynthesis genes in the genus *Bradyrhizobium *.

## Phenotypic characterization

Novel strains 65S1MB, 85S1MB^T^ and 101S1MB exhibit colonies that are circular, convex, beige, translucent and <1 mm diameter after 7 days growth at 28 °C on yeast extract–mannitol (YEM) agar medium [2]. Bacterial cells are Gram-stain-negative based on the KOH method of Buck [29]. Production of an alkaline reaction on YEM agar after 21 days growth at 28 °C (Table S2) is typical of the genus *Bradyrhizobium *. Cell morphology was investigated using a transmission electron microscope (H-700, Hitachii) as described previously [4]. Cells are rod-shaped with sub-polar flagella and have average cell sizes (Fig. S10) consistent with the characteristics of the genus *Bradyrhizobium* [30].

Analysis of fatty acids was done using the Sherlock Microbial Identification System (midi) version 6.0 and the rtsba6 database as described by Yu *et al.* [4]. The novel strains exhibited fatty acid profiles (Table S3) characteristic of the genus *Bradyrhizobium* [31] with a predominance of fatty acids C_16 : 0_ and C_18__ : __1_ω6*c*/C_18__ : __1_ω7*c* (summed feature 8).

Various phenotypic tests, including 70 carbon source utilization and 18 chemical sensitivity assays, were done using Biolog GEN III MicroPlates according to manufacturer's instructions. The results (Table S2) showed that novel strains 65S1MB, 85S1MB^T^ and 101S1MB could be differentiated from close relatives, *B. amphicarpaeae* 39S1MB^T^, *B. ottawaense* OO99^T^ and *B. shewense* ERR11^T^ as well as from *B. japonicum* USDA6^T^, *B. betae* PL7HG1^T^ and *B. diazoefficiense* USDA110^T^ on the basis of several of these phenotypic assays.

Plant tests using Leonard jars (three replicate jars, two plants per jar) were carried out as described previously [2]. Results of tests done in this work and in the previous study [3], confirmed that the novel strains do not elicit nodules on roots of soybean ‘AC Glengarry’, *Macroptilium atropurpureum* ‘Siratro’ or on roots of three legumes native to east Canada (*Amphicarpaea bracteata*, *Desmodium canadense* and *Desmodium glutinosum*).

Based on the phylogenetic, complete genome sequence and phenotypic data presented here, we propose that the four novel strains (65S1MB, 85S1MB^T^, 101S1MB and 141S2) represent a novel species named *Bradyrhizobium symbiodeficiens* sp. nov.

## Description of *Bradyrhizobium symbiodeficiens* sp. nov.

*Bradyrhizobium symbiodeficiens* (sym.bi.o.de.fi′ci.ens. Gr. masc. adj. *symbios* living together; L. v. *deficio* to fail, to be wanting to; N.L. part. adj. *symbiodeficiens* deficient of symbiosis).

Cells are Gram-stain-negative, aerobic, non-spore-forming rods (approx. 1.75 µm long x0.83 µm wide) with sub-polar flagella. Colonies on YEM agar medium are circular, convex, beige, translucent and <1 mm in diameter after 7 days at 28 °C. Growth occurs at pH 5 and pH 10 (optimum, pH 7.0). Produces an alkaline reaction on YEM agar. The type strain grows at 10 °C, optimal at 28 °C, but no growth occurs at 37 °C. Grows in the presence of 1 % (w/v) NaCl. Utilizes l-fucose, l-pyroglutamic acid, l-galactonic acid lactone, d-gluconic acid, p-hydroxyphenylacetic acid, α-keto-glutaric acid, d-malic acid, l-malic acid, bromo-succinic acid, propionic acid, acetic acid and nine other carbon sources. Does not utilize d-mannose, d-mannitol, d-arabitol, l-aspartic acid, l-glutamic acid, mucic acid, quinic acid, d-saccharic acid, citric acid, Tween 40, γ-amino-butryric acid, α-hydroxybutyric acid, acetoacetic acid and 38 other carbon sources. Variable result with d-galactose. Resistant to troleandomycin, rifamycin SV, minocycline, vancomycin, tetrazolium violet, tetrazolium blue, potassium tellurite, aztreonam and three other chemical compounds. Susceptible to sodium butyrate and five other chemical compounds. Variable result with fusidic acid.

Predominant fatty acids are C_16__ : __0_ and C_18__ : __1_ω6*c*/C_18__ : __1_ω7*c* (summed feature 8). Does not elicit root nodules on *Glycine max*, *Macroptilium atropurpureum*, *Amphicarpaea bracteata*, *Desmodium canadense* and *Desmodium glutinosum*.

The type strain, 85S1MB^T^ (=LMG 29937^T^=HAMBI 3684^T^), was isolated from a root nodule of a soybean plant that was inoculated with root-zone soil of *Amphicarpaea bracteata* (Hog peanut) growing in Canada. The type strain does not contain photosystem genes, type III secretion system genes or key nodulation and nitrogen-fixation genes. The DNA G+C content of the type strain is 64.3 mol% and the genome size is 7.04 Mbp. GenBank/EMBL/DDBJ accession numbers for the complete genome and the 16S rRNA, *atp* *D*, *gln* *II*, *rec* *A*, *gyr* *B* and *rpo* *B* gene sequences of the type strain are CP029427, KP768783, KP768551, KP768609, KF615036, KP768725 and KP768667, respectively.

## Supplementary Data

Supplementary material 1
