# Supplementary material for: Description and complete genome sequences of Bradyrhizobium symbiodeficiens sp. nov., a non-symbiotic bacterium associated with legumes native to Canada
Source: Int J Syst Evol Microbiol. 2020 Feb 3;70(1):442–9. doi: 10.1099/ijsem.0.003772 (PMC7395627; doi:10.1099/ijsem.0.003772)
Supplement: Supplementary material 1 [file ijsem-70-442-s001.pdf]

Supplementary Table S1. GenBank nucleotide accession numbers for strains of *B. symbiodeficiens* sp. nov. and reference taxa.

Strain	*atpD*	*glnII*	*gyrB*	*recA*	*rpoB*	*16S rRNA*
***B. symbiodeficiens* 85S1MB^T**	KP768551	KP768609	KP768725	KF615036	KP768667	KP768783
***B. symbiodeficiens* 65S1MB**	KP768549	KP768607	KP768723	KF615024	KP768665	KP768781
***B. symbiodeficiens* 101S1MB**	KP768554	KP768612	KP768728	KF615048	KP768670	KP768786
***B. symbiodeficiens* 141S2**	KP768565	KP768623	KP768739	KF615231	KP768681	KP768797
B. algeriense RST89^T	PYCM01000338	PYCM01000763	PYCM01000687	PYCM01000643	PYCM01000653	FJ546419
B. americanum CMVU44^T	-	KX012942	NA	KC247141	NA	KU991833
B. amphicarpaeae 39S1MB^T	KP768547	KP768605	KF615002	KP768721	KP768663	KP768779
B. arachidis CCBAU051107^T	KF962683	KF962689	KF962693	KF962707	JX437682	HM107167
B. betae LMG21987^T	FM253129	AB353733	FM253217	AB353734	FM253260	NR_029104
B. brasilense UFLA03-321^T	KF452730	MPVQ00000000	KF452827	KT793142	MPVQ00000000	MPVQ00000000
B. cajani AMBPC1010^T	NA	KY349442	NA	KY349440	NA	KY349447
B. canariense LMG22265^T	FM253135	AY386765	FM253220	AY591553	FM253263	AJ558025
B. centrolobii BR10245^T	LUUB01000107	LUUB01000131	LUUB01000063	LUUB01000078	LUUB01000064	KF927049
B. centrosemae A9^T	-	KX012940	NA	KC247145	NA	KC247115
B. cytisi CTAW11^T	JN186289	JN186291	JN186292	JN186293	JN186288	EU561065
B. daqingense CCBAU15774^T	HQ231289	KF962690	KF962694	KF962708	JX437676	KJ184551
B. denitrificans LMG8443^T	FM253153	HM047121	FM253239	EU665419	FM253282	NR_041827
B. diazoefficiense USDA 110	BA000040	BA000040	BA000040	BA000040	BA000040	BA000040
B. elkanii USDA76^T	AY386758	AY599117	AM418800	AY591568	EF190188	HQ233240
B. embrapense CNPSo2833^T	LFIP02000012	LFIP0200001	LFIP0200010	LFIP0200009	LFIP0200004	AY904773
B. erythrophlei CCBAU53325^T	NA	KF114693	-	KF114669	-	KF1144645
B. ferriligni CCBAU51502^T	NA	KJ818099	-	KJ818112	-	KJ818096
B. forestalis INPA54B^T	PGVG00000000	KF452867	PGVG00000000	PGVG00000000	PGVG00000000	PGVG00000000
B. ganzhouense RITF806^T	-	JX277110	NA	JX277144	NA	JQ796661
B. guangdongense CCBAU51649^T	-	KC509023	-	KC509269	-	KC508867
B. guangxiense CCBAU53363^T	-	KC509033	-	KC509279	-	KC508877
B. huanghuaihaiense CCBAU23303^T	HQ231682	KF962691	KF962695	KF962709	JX437679	HQ231463
B. icense LMTR13^T	KF896192	KF896175	KF896201	JX943615	CP016428	KF896156
B. ingae BR10250^T	-	KF927067	-	KF927061	-	KF927043
B. iriomotense EK05^T	AB300994	AB300995	HQ873308	AB300996	HQ587646	AB300992

Strain	*atpD*	*glnII*	*gyrB*	*recA*	*rpoB*	*16S rRNA*
B. japonicum USDA6^{T}	AM168320	HQ587875	AM418801	AM168341	AY242830	AB510002
B. jicamae LMG24556^{T}	FJ428211	FJ428204	HQ873309	HQ587415	HQ587647	AY624134
B. kavangense 14-3^{T}	KY753592	KM378446	KX661397	KM378399	KM378311	KP899562
B. lablabi CCBAU23086 ^{T}	GU433473	GU433498	KF962696	KF962710	JX437677	GU433448
B. liaoningense LMG18230^{T}	FM253137	AY386775	FM253223	AY591564	FM253266	AJ250813
B. lupini USDA3051^{T}	-	KM114862	-	KM114866	NA	KM114861
B. macuxiense BR10303^{T}	LNCU01000024	LNCU01000062	LNCU01000041	LNCU01000014	LNCU01000011	KX527919
B. manausense BR3351^{T}	LJYG01000004	LJYG01000112	LJYG01000105	LJYG01000054	LJYG01000045	HQ641226
B. mercantei SEMIA 6399^{T}	MKFI00000000	KX690621	KX690617	KX690615	MKFI00000000	FJ025102
B. namibiense 5-10^{T}	KX661387	KM378440	KX661393	KM378377	KM378306	KX661401
B. neotropical BR10247^{T}	LSEF00000000	KJ661700	LSEF00000000	KF785992	KF983829	KF927051
B. nitroreducens TSA1^{T}	LFJC00000000	LFJC00000000	LFJC00000000	LFJC00000000	LFJC00000000	LFJC00000000
B. oligotrophicum S58^{T}	JQ619232	JQ619233	KF962697	JQ619231	KF962713	JQ619230
B. ottawaense OO99^{T}	HQ455212	HQ587750	HQ873179	HQ587287	HQ587518	JN186270
B. pachyrhizi LMG24246^{T}	FJ428208	FJ428201	HQ873310	HQ587416	HQ587648	AY624135
B. paxllaeri LMTR21^{T}	CP042968	CP042968	CP042968	CP042968	CP042968	AY923031
B. retamae Ro19 ^{T}	KC247101	KC247108	KF962698	KF962711	KF962714	KC247085
B. rifense CTAW71^{T}	GU001617	KF962692	KF962699	KF962712	KF962715	EU561074
B. ripae WR4^{T}	NA	MF593086	-	MF593090	-	MF593082
B. sacchari BR10280^{T}	KX065107	KX065099	LWIG00000000	KX065095	LWIG00000000	KF113091
B. shewense ERR11^{T}	FMAI00000000	FMAI00000000	FMAI00000000	FMAI00000000	FMAI00000000	FMAI00000000
B. stylosanthis BR446^{T}	LVEM01000002	LVEM01000001	LVEM01000002	LVEM01000001	LVEM01000001	KU724142
B. subterraneum 58-2-1^{T}	-	KM378484	-	KM378397	-	KP308152
B. tropiciagri CNPSo1112^{T}	LFLZ01000050	LFLZ01000067	LFLZ01000066	LFLZ01000039	LFLZ01000008	AY904753
B. valentinum LmjM3^{T}	LLXX01000203	LLXX01000118	LLXX01000044	LLXX01000017	LLXX01000029	JX514883
B. vignae 7-2^{T}	RDQF01000079	RDQF01000005	RDQF01000001	RDQF01000045	RDQF01000055	KP899563
B. viridifuturi SEMIA690^{T}	LGTB01000039	LGTB01000012	LGTB01000021	LGTB01000025	LGTB01000001	FJ025107
B. yuanmingense CCBAU10071^{T}	FM253140	AY386780	FM253226	AM168343	FM253269	AB509380

NA Sequence not available in public databases.

- Sequence not used in phylogenetic analyses of five housekeeping genes.

Supplementary Table S2. Phenotypic characteristics of **1**. *B. symbiodeficiens* sp. nov. 85S1MB^T, **2**. *B. symbiodeficiens* 65S1MB, **3**. *B. symbiodeficiens*, 101S1MB, **4**. *B. amphicarpaeae* 39S1MB^T, **5**. *B. ottawaense* OO99^T, **6**. *B. shewense* ERR11^T, **7**. *B. japonicum* USDA6^T, **8**. *B. betae* PL7HG1^T, **9**. *B. diazoefficiense* USDA110^T.

**Characteristic**	**1**	**2**	**3**	**4**	**5**	**6**	**7**	**8**	**9**	**Characteristic**	**1**	**2**	**3**	**4**	**5**	**6**	**7**	**8**	**9**
C-source utilization (Biolog)[a]																			
Dextrin	-	-	-	-	-	-	-	-	-	Glycyl-L-Proline	-	-	-	-	-	-	-	-	-
D-Maltose	-	-	-	-	-	-	-	-	-	L-Alanine	-	-	-	-	-	-	-	-	-
D-Trehalose	-	-	-	-	-	-	-	-	-	L-Arginine	-	-	-	-	-	-	-	-	-
D-Cellobiose	-	-	-	-	-	-	-	-	-	L-Aspartic Acid	-	-	-	-	-	+	-	-	-
Gentiobiose	-	-	-	-	-	-	-	-	-	L-Glutamic Acid	-	-	-	-	-	+	-	-	±
Sucrose	-	-	-	-	-	-	-	-	-	L-Histidine	-	-	-	-	-	-	-	-	-
D-Turanose	-	-	-	-	-	-	-	-	-	L-Pyroglutamic Acid	+	+	+	+	-	+	-	-	+
Stachyose	-	-	-	-	-	-	-	-	-	L-Serine	-	-	-	-	-	-	-	-	-
D-Raffinose	-	-	-	-	-	-	-	-	-	Pectin	-	-	-	-	-	-	-	-	-
α-D-Lactose	-	-	-	-	-	-	-	-	-	D-Galacturonic Acid	+	+	+	+	+	+	+	+	+
D-Melibiose	-	-	-	-	-	-	-	-	-	L-Galactonic Acid Lactone	+	+	+	+	+	±	-	+	+
β-Methyl-DGlucoside	-	-	-	-	-	-	-	-	-	D-Gluconic Acid	+	+	+	+	+	+	-	-	+
D-Salicin	-	-	-	-	-	-	-	-	-	D-Glucuronic Acid	+	+	+	+	+	+	+	+	+
N-Acetyl-DGlucosamine	-	-	-	-	-	-	-	-	-	Glucuronamide	+	+	+	+	+	+	+	+	+
N-Acetyl-β-DMannosamine	-	-	-	-	-	-	-	-	-	Mucic Acid	-	-	-	-	+	+	+	-	+
N-Acetyl-DGalactosamine	-	-	-	-	-	-	-	-	-	Quinic Acid	-	-	-	+	±	±	±	-	±
N-Acetyl Neuraminic Acid	-	-	-	-	-	-	-	-	-	D-Saccharic Acid	-	-	-	-	+	+	+	-	+
α-D-Glucose	-	-	-	-	-	-	-	-	-	p-HydroxyPhenylacetic Acid	+	±	±	-	-	-	-	±	-
D-Mannose	-	-	-	-	-	±	-	-	-	Methyl Pyruvate	+	+	+	+	+	+	+	±	+
D-Fructose	-	-	-	-	-	-	-	-	-	D-Lactic Acid Methyl Ester	-	-	-	-	-	-	-	-	-
D-Galactose	±	-	-	-	-	±	-	-	±	L-Lactic Acid	+	+	+	+	+	+	+	+	+
3-Methyl Glucose	-	-	-	-	-	-	-	-	-	Citric Acid	-	-	-	-	±	+	-	-	-
D-Fucose	+	+	+	+	+	+	+	+	+	α-Keto-Glutaric Acid	+	+	+	+	+	-	-	±	+
L-Fucose	±	±	±	±	-	+	-	-	±	D-Malic Acid	+	+	+	-	±	+	+	-	+
L-Rhamnose	-	-	-	-	-	-	-	-	-	L-Malic Acid	+	+	+	+	+	+	+	±	+
Inosine	-	-	-	-	-	-	-	-	-	Bromo-Succinic Acid	±	±	±	-	-	+	-	-	+
D-Sorbitol	-	-	-	-	-	-	-	-	-	Tween 40	-	-	-	-	-	+	-	±	±
D-Mannitol	-	-	-	±	-	+	-	-	-	γ-Amino-Butryric Acid	-	-	-	-	-	-	-	-	-
D-Arabitol	-	-	-	±	+	+	-	-	-	α-HydroxyButyric Acid	-	-	-	-	-	-	-	±	±
myo-Inositol	-	-	-	-	-	-	-	-	-	β-Hydroxy-D,LButyric Acid	+	+	+	+	+	+	+	+	+
Glycerol	+	+	+	+	+	+	+	±	+	α-Keto-Butyric Acid	-	-	-	-	-	-	-	-	-
D-Glucose- 6-PO4	-	-	-	-	-	-	-	-	-	Acetoacetic Acid	-	-	-	-	-	-	-	+	-
D-Fructose- 6-PO4	-	-	-	-	-	-	-	-	-	Propionic Acid	+	+	+	±	-	-	-	-	+
D-Aspartic Acid	-	-	-	-	-	-	-	-	-	Acetic Acid	+	+	+	+	-	±	-	-	+
D-Serine	-	-	-	-	-	-	-	-	-	Formic Acid	+	+	+	+	+	+	+	+	+
Gelatin	-	-	-	-	-	-	-	-	-										
Chemical Sensitivity (Biolog)[a]																			
1% Sodium Lactate	+	+	+	+	+	+	+	±	±	Vancomycin	+	+	+	±	-	-	+	±	-
Fusidic Acid	-	+	-	-	-	-	-	-	-	Tetrazolium Violet	+	+	+	-	+	+	+	+	±
D-Serine	-	-	-	-	-	-	-	-	-	Tetrazolium Blue	+	+	+	-	+	+	+	+	+
Troleandomycin	+	+	+	+	±	-	-	+	+	Nalidixic Acid	+	+	+	+	+	±	+	±	±
Rifamycin SV	+	+	+	+	-	-	+	±	+	Lithium Chloride	-	-	-	-	-	-	-	-	-
Minocycline	+	+	+	+	+	-	+	+	+	Potassium Tellurite	+	+	+	-	-	-	-	±	±
Lincomycin	+	+	+	+	±	±	+	±	±	Aztreonam	+	+	+	+	±	-	+	+	-
Guanidine HCl	-	-	-	-	-	-	-	-	-	Sodium Butyrate	-	-	-	-	-	-	-	±	-
Niaproof 4	-	-	-	-	-	-	-	-	-	Sodium Bromate	-	-	-	-	-	-	-	-	-
Growth on YEM agar medium:																			
10 °C	±	±	±	-	-	ND	ND	ND	ND										
37 °C	-	-	-	-	-	-	-	-	-										
pH 5	+	+	+	+	+	+	+[b]	+[b]	+										
pH 10	±	±	±	±	±	+	ND	ND	+										
1% NaCl	±	±	±	-	-	-	+[c]	+[c]	-										
Acid/alkali production (pH)[d]	8.0	8.5	8.0	8.5	8.0	8.0	7.8	ND	ND										

[a] BIOLOG GEN III MicroPlates (120 hours incubation at 28 ºC): +, Positive; ±, weak; −, negative. ND, not determined. Values are based on three replicates.

[b] From: Delamuta JR, Ribeiro RA, Ormeño-Orrillo E, Melo IS, Martínez-Romero E, *et al. Int J Syst Evol Microbiol* 2013; 63: 3342–3351.

[c] From: Rivas R, Willems A, Palomo JL, Garcıa-Benavides P, Mateos PF, ***et al.*** *Int J Syst Evol Microbiol* 2004; 54: 1271–1275.

[d] Acid/alkali production after 21 days at 28 C; uninoculated control, pH 7.1.

Supplementary Table S3. Fatty acid patterns of *Bradyrhizobium* strains: **1**. *B. symbiodeficiens* sp. nov. 85S1MB^T, **2**. *B. symbiodeficiens,* 65S1MB, **3**. *B. symbiodeficiens* 101S1MB, **4**. *B. amphicarpaeae* 39S1MB^T; **5**. *B. ottawaense* OO99^T, **6**. *B. japonicum* USDA6^T and **7**. *B. diazoefficiense* USDA110^T.

**Fatty Acid**	**1**	**2**	**3**	**4***	**5†**	**6†**	**7‡**
11:0	-	-	-	0.7	-	-	-
12:1	-	-	-	0.9	-	-	-
16:0	6.3	5.9	6.3	9.7	7.9	13.1	14.1
16:1 ω5c	7.7	9.5	6.7	6.3	6.3	3.6	-
17:1 iso 1ω5c	-	-	-	1.0	-	-	-
17:1 anteiso 1ω9c	-	-	-	2.0	-	-	-
17:0 anteiso	-	-	-	0.4	-	-	-
17:1 ω8c	1.1	1.8	1.1	2.1	0.8	-	-
17:1 ω6c	0.6	1.0	0.6	2.0	-	-	-
17:0	0.4	-	0.5	1.2	0.4	-	-
18:0	0.3	-	0.5	0.7	0.7	0.8	-
18:1 ω5c	0.3	-	-	-	-	-	-
C18:1 ω7c 11-methyl	0.5	-	0.5	-	-	6.7	-
19:0 cyclo ω8c	0.3	-	1.3	-	-	-	-
Summed feature 3§	3.3	4.0	2.8	1.9	1.6	1.1	-
Summed feature 7§	1.0	-	0.8	0.6	-	-	-
Summed feature 8§	78.2	77.9	78.9	70.4	82.3	74.8	85.9

- Not detected.

* Data from: Bromfield ESP, Cloutier S, Nguyen HDT. *Int J Syst Evol Microbiol* 2019; DOI: 10.1099/ijsem.0.003569.

† Data from: Yu X, Cloutier S, Tambong J, Bromfield ESP. *Int J Syst Evol Microbiol* 2014; 64: 3202–3207.

‡ Data from: Delamuta JR, Ribeiro RA, Ormeño-Orrillo E, Melo IS, Martínez-Romero E *et al. Int J Syst Evol Microbiol* 2013; 63: 3342-3351.

§ Summed features represent groups of fatty acids that were not separated by GLC with the MIDI system. Summed feature 3, 16:1 ω6c/16:1 ω7c; Summed feature 7, 19:1 ω7c/19:1 ω6c; summed feature 8, 18:1 ω6c/18:1 ω7c.

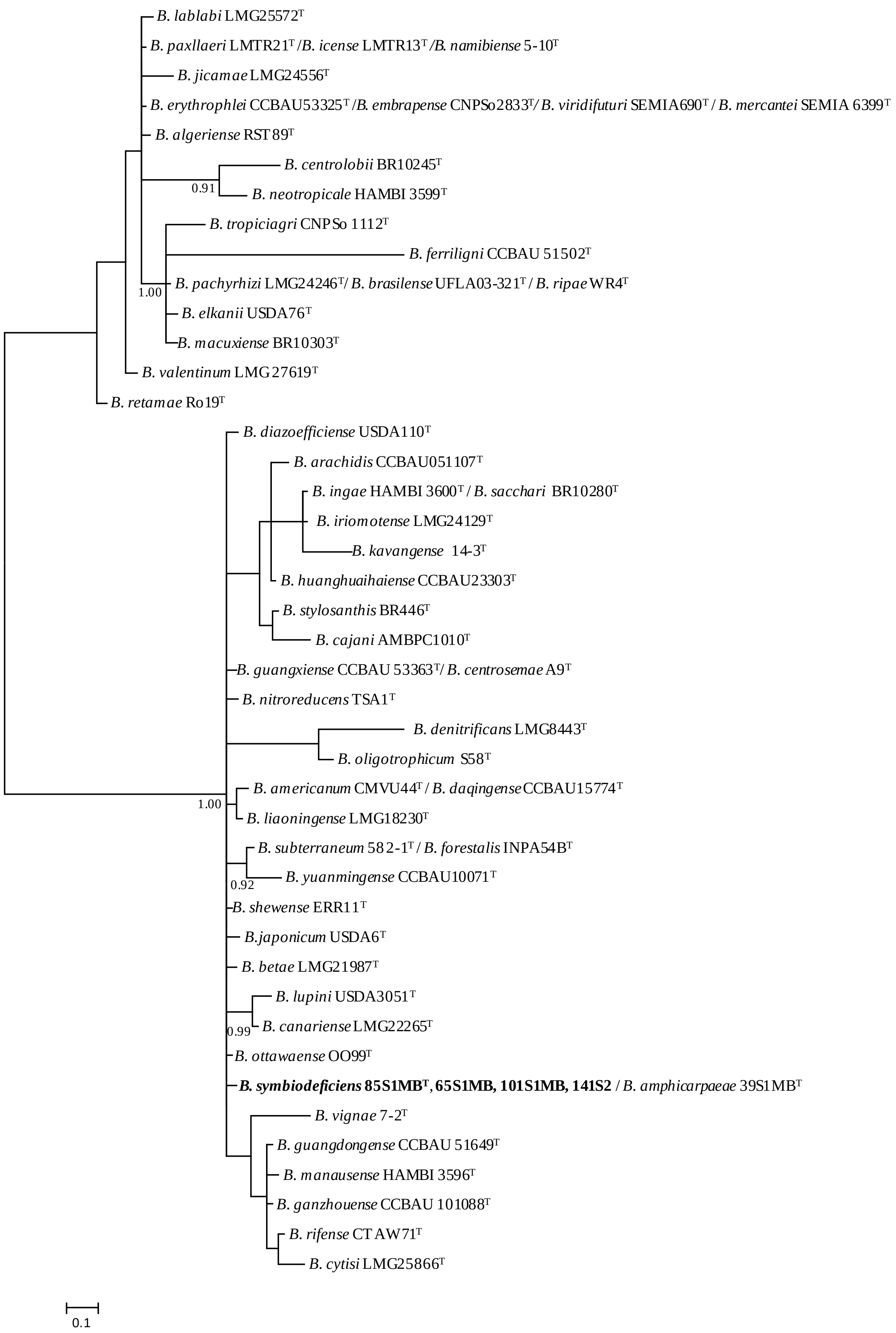
B. lablabi LMG25572T
B. paxllaeri LMTR21T /B. icense LMTR13T /B. namibiense 5-10T
B. jicamae LMG24556T
B. erythrophlei CCBAU53325T /B. embrapense CNPSo2833T/ B. viridifuturi SEMIA690T / B. mercantei SEMIA 6399T
B. algeriense RST 89T
B. centrolobii BR10245T
0.91
B. neotropicale HAMBI 3599T
B. tropiciagri CNPSo 1112T
B. ferriligni CCBAU 51502T
B. pachyrhizi LMG24246T/ B. brasilense UFLA03-321T / B. ripae WR4T
1.00
B. elkanii USDA76T
B. macuxiense BR10303T
B. valentinum LMG 27619T
B. retamae Ro19T
B. diazoefficiense USDA110T
B. arachidis CCBAU051107T
B. ingae HAMBI 3600T / B. sacchari BR10280T
B. iriomotense LMG24129T
B. kavangense 14-3T
B. huanghuaihaiense CCBAU23303T
B. stylosanthis BR446T
B. cajani AMBPC1010T
B. guangxiense CCBAU 53363T/ B. centrosemae A9T
B. nitroreducens TSA1T
B. denitrificans LMG8443T
B. oligotrophicum S58T
B. americanum CMVU44T / B. daqingense CCBAU15774T
1.00
B. liaoningense LMG18230T
B. subterraneum 58 2-1T / B. forestalis INPA54BT
B. yuanmingense CCBAU10071T
0.92
B. shewense ERR11T
B.japonicum USDA6T
B. betae LMG21987T
B. lupini USDA3051T
B. canariense LMG22265T
0.99
B. ottawaense OO99T
B. symbiodeficiens 85S1MBT, 65S1MB, 101S1MB, 141S2 / B. amphicarpaeae 39S1MBT
B. vignae 7-2T
B. guangdongense CCBAU 51649T
B. manausense HAMBI 3596T
B. ganzhouense CCBAU 101088T
B. rifense CTAW71T
B. cytisi LMG25866T
0.1

Supplementary Fig. S1 Bayesian phylogenetic tree of 16S rRNA gene sequences for *Bradyrhizobium symbiodeficiens* sp. nov., and 54 reference taxa (HKY + G + I substitution model). Only posterior probabilities ≥ 90% are shown. Scale bar represents expected number of substitutions per site.

To include all named species of *Bradyrhizobium* in the tree, alignment lengths of 16S rRNA gene sequences were trimmed to 1238 bp.

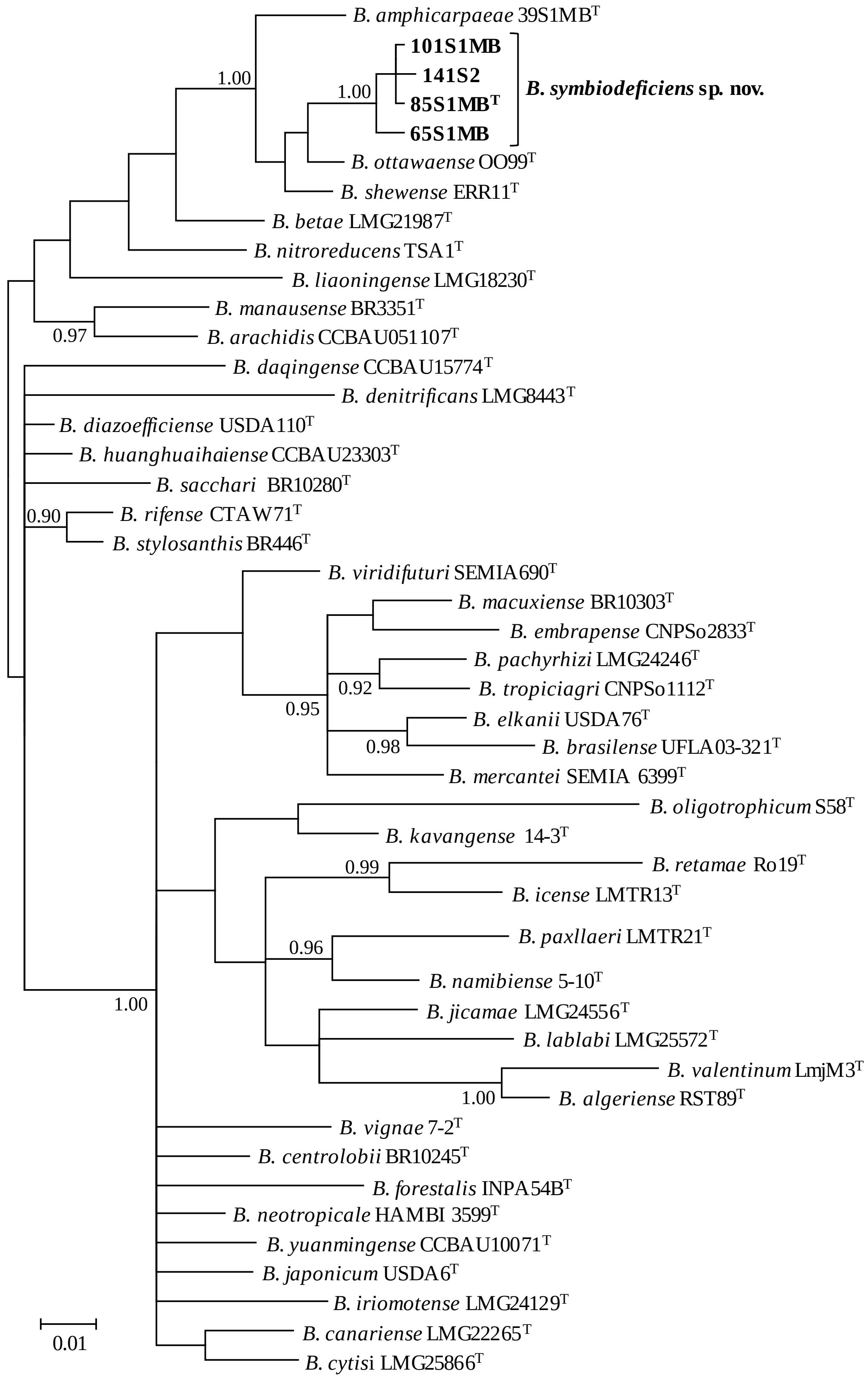

Fig. S2. Bayesian phylogenetic tree (GTR + G + I substitution model) of *atpD* housekeeping gene sequences (429 bp) for *Bradyrhizobium symbiodeficiens* sp. nov. and reference taxa of the genus *Bradyrhizobium*. Posterior probabilities ≥ 90% are shown. Bar, expected substitutions per site.

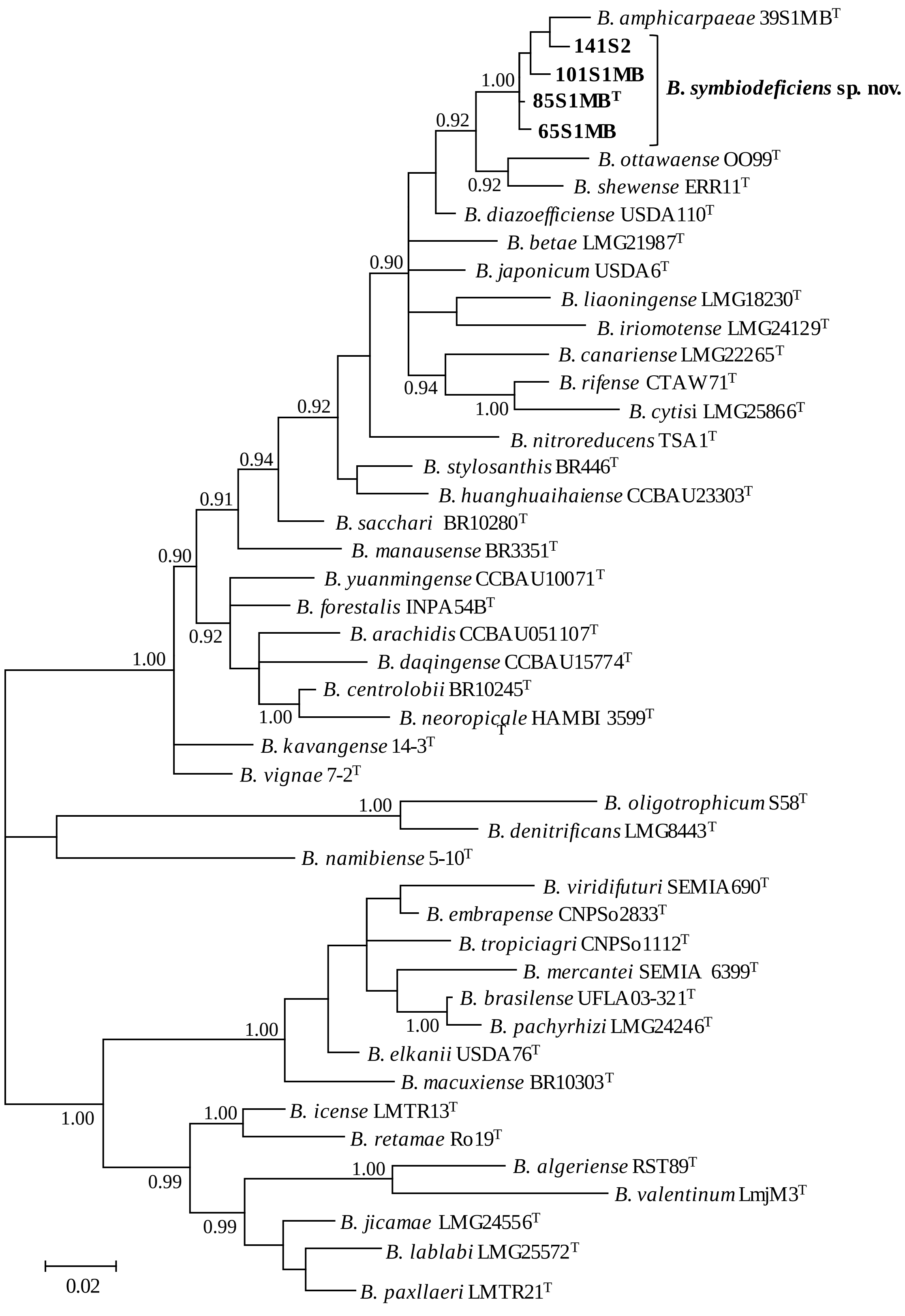

Fig. S3. Bayesian phylogenetic tree (GTR + G + I substitution model) of *gln*II housekeeping gene sequences (519 bp) for *Bradyrhizobium symbiodeficiens* sp. nov. and reference taxa of the genus *Bradyrhizobium*. Posterior probabilities ≥ 90% are shown. Bar, expected substitutions per site.

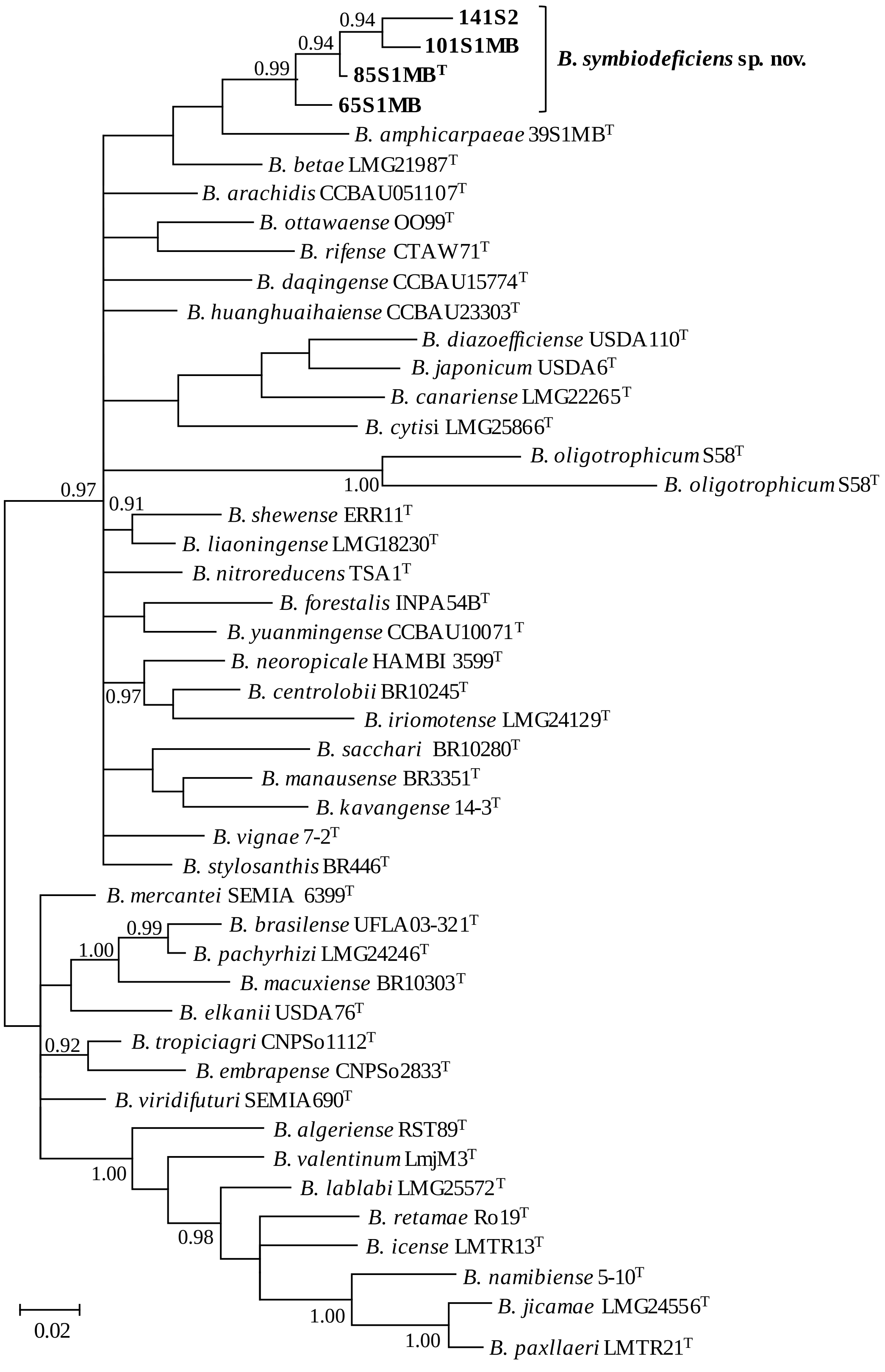

Fig. S4. Bayesian phylogenetic tree (GTR + G + I substitution model) of *recA* housekeeping gene sequences (417 bp) for *Bradyrhizobium symbiodeficiens* sp. nov. and reference taxa of the genus *Bradyrhizobium*. Posterior probabilities ≥ 90% are shown. Bar, expected substitutions per site.

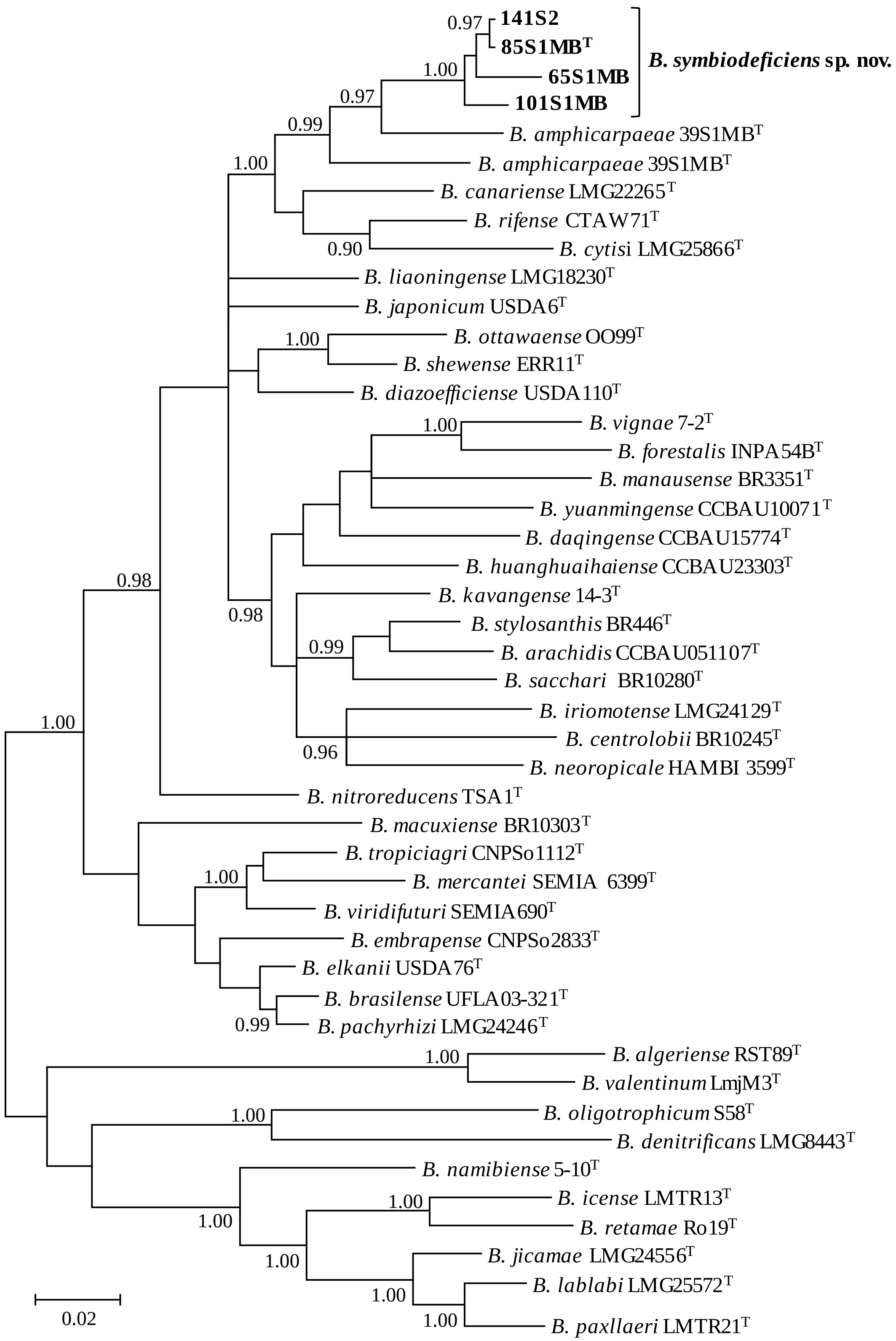

Fig. S5. Bayesian phylogenetic tree (GTR + G + I substitution model) of *gyrB* housekeeping gene sequences (600 bp) for *Bradyrhizobium symbiodeficiens* sp. nov. and reference taxa of the genus *Bradyrhizobium*. Posterior probabilities ≥ 90% are shown. Bar, expected substitutions per site.

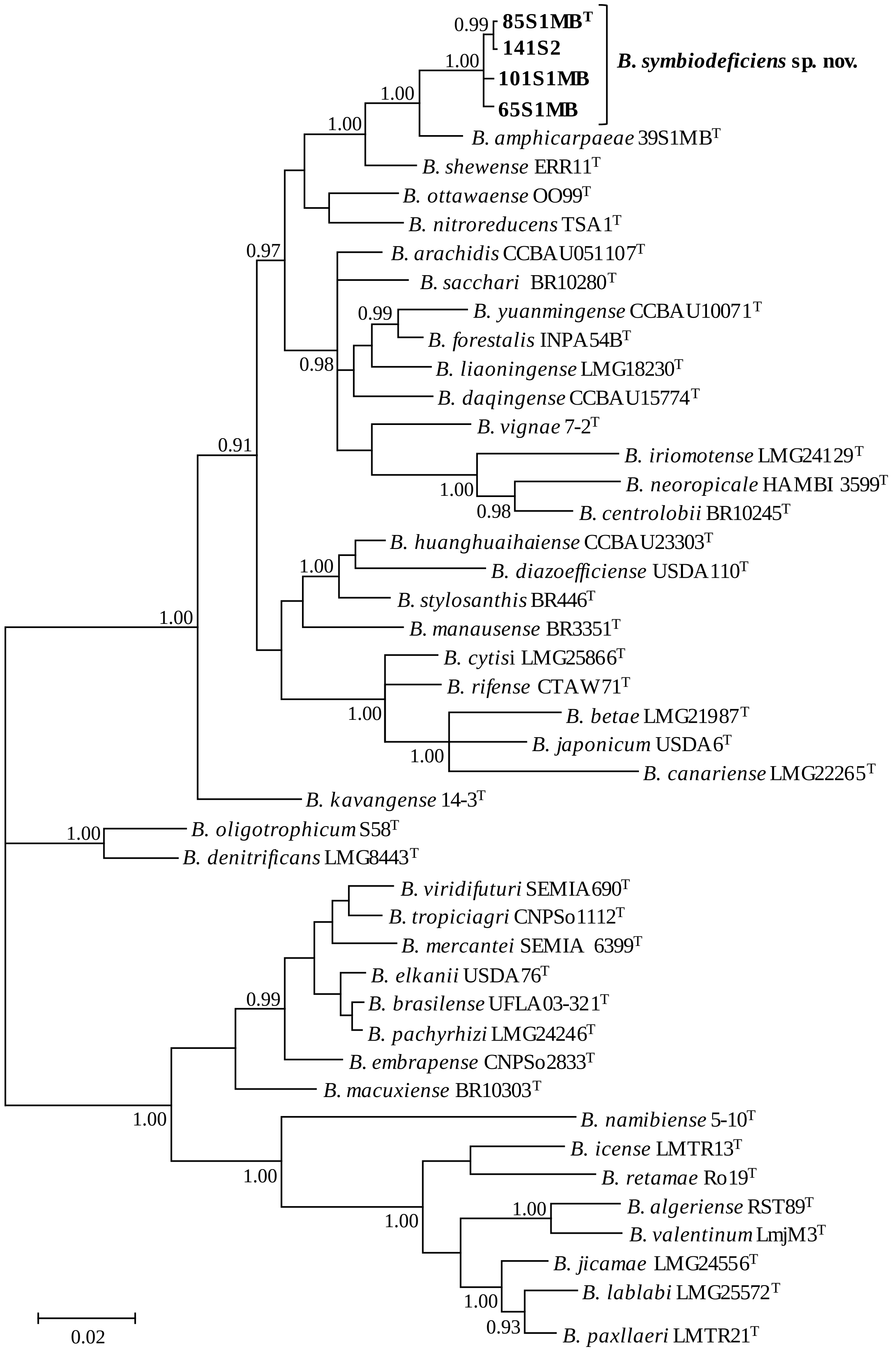

Fig. S6. Bayesian phylogenetic tree (GTR + G + I substitution model) of *rpoB* housekeeping gene sequences (714 bp) for *Bradyrhizobium symbiodeficiens* sp. nov. and reference taxa of the genus *Bradyrhizobium*. Posterior probabilities ≥ 90% are shown. Bar, expected substitutions per site.

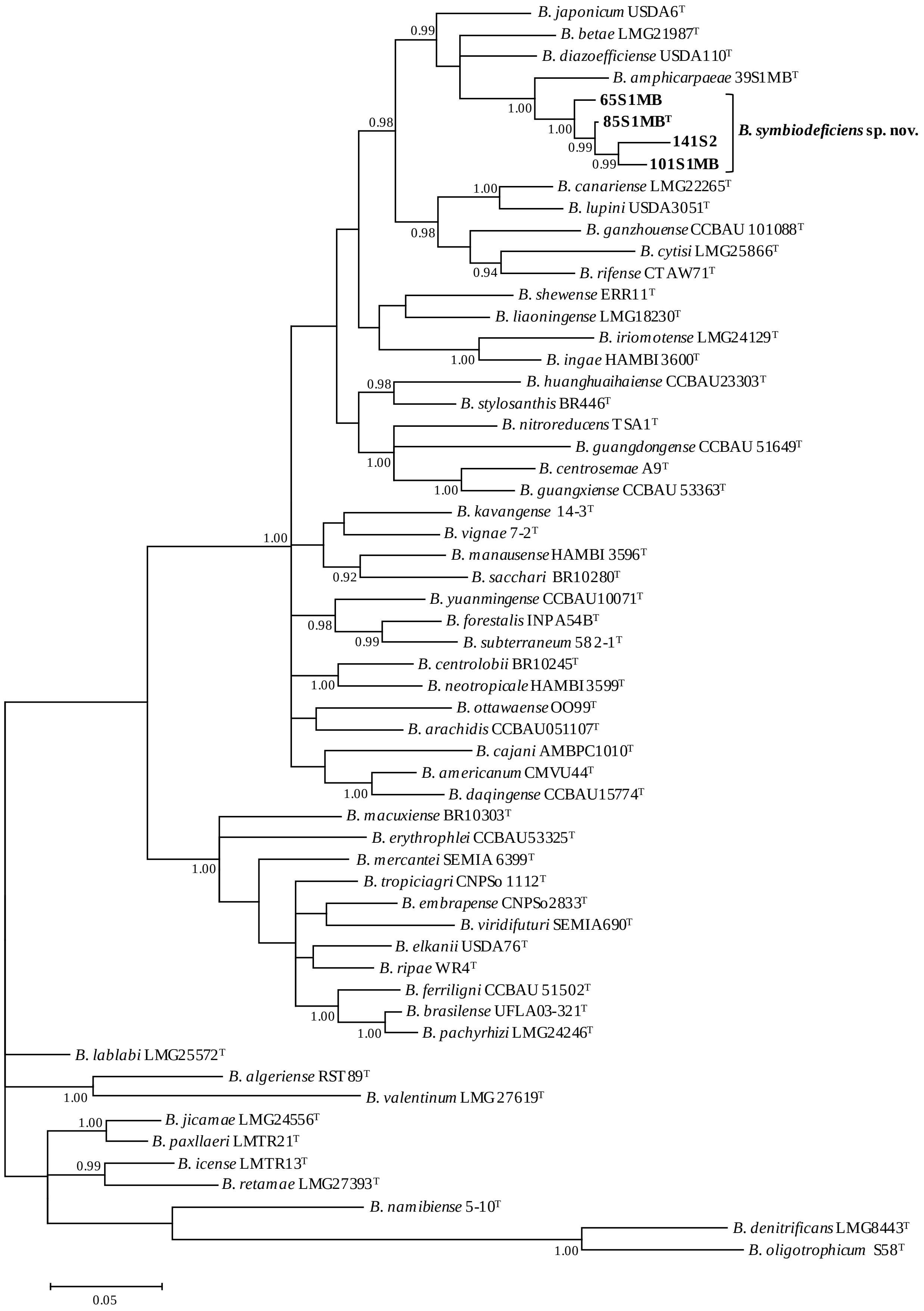

B. japonicum USDA6^{T}
B. betae LMG21987^{T}
B. diazoefficiense USDA110^{T}
B. amphicarpaeae 39S1MB^{T}
65S1MB
85S1MB^{T}
141S2
101S1MB
B. symbiodeficiens sp. nov.
B. canariense LMG22265^{T}
B. lupini USDA3051^{T}
B. ganzhouense CCBAU 101088^{T}
B. cytisi LMG25866^{T}
B. rifense CTAW71^{T}
B. shewense ERR11^{T}
B. liaoningense LMG18230^{T}
B. iriomotense LMG24129^{T}
B. ingae HAMBI 3600^{T}
B. huanghuaihaiense CCBAU23303^{T}
B. stylosanthis BR446^{T}
B. nitroreducens TSA1^{T}
B. guangdongense CCBAU 51649^{T}
B. centrosemae A9^{T}
B. guangxiense CCBAU 53363^{T}
B. kavangense 14-3^{T}
B. vignae 7-2^{T}
B. manausense HAMBI 3596^{T}
B. sacchari BR10280^{T}
B. yuanmingense CCBAU10071^{T}
B. forestalis INPA54B^{T}
B. subterraneum 58 2-1^{T}
B. centrolobii BR10245^{T}
B. neotropicale HAMBI 3599^{T}
B. ottawaense OO99^{T}
B. arachidis CCBAU051107^{T}
B. cajani AMBPC1010^{T}
B. americanum CMVU44^{T}
B. daqingense CCBAU15774^{T}
B. macuxiense BR10303^{T}
B. erythrophlei CCBAU53325^{T}
B. mercantei SEMIA 6399^{T}
B. tropiciagri CNPSo 1112^{T}
B. embrapense CNPSo2833^{T}
B. viridifuturi SEMIA690^{T}
B. elkanii USDA76^{T}
B. ripae WR4^{T}
B. ferriligni CCBAU 51502^{T}
B. brasilense UFLA03-321^{T}
B. pachyrhizi LMG24246^{T}
B. lablabi LMG25572^{T}
B. algeriense RST89^{T}
B. valentinum LMG 27619^{T}
B. jicamae LMG24556^{T}
B. paxllaeri LMTR21^{T}
B. icense LMTR13^{T}
B. retamae LMG27393^{T}
B. namibiense 5-10^{T}
B. denitrificans LMG8443^{T}
B. oligotrophicum S58^{T}
0.05

Supplementary Fig. S7. Bayesian phylogenetic tree (GTR + G + I substitution model) of *rec*A-*gln*II concatenated gene sequences (854 bp) for *Bradyrhizobium symbiodeficiens* sp. nov. and reference taxa of the genus *Bradyrhizobium*. Only posterior probabilities ≥ 90% are shown. Bar, expected substitutions per site. To include all named species of *Bradyrhizobium* in the analysis, it was necessary to trim individual sequence alignment lengths to 375 and 479 bp for *rec*A and *gln*II, respectively.

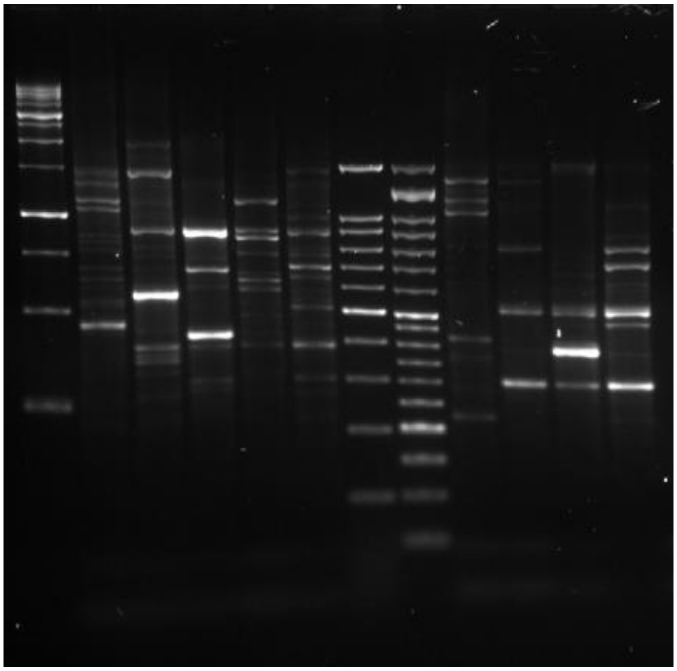

Supplementary Fig. S8. Example of RAPD fingerprint profiles generated by one of four random primers (primer P5). PCR products were separated on 2% agarose for 2 hrs at 4 V cm-1.
Lanes: 1, *B. liaoningense* LMG18230^{T}; 2, *B. daqingense* CCBAU15774^{T}; 3, *B. huanghuaihaiense* CCBAU23303^{T}; 4, *B. diazoefficiens* USDA110^{T}; 5, *B. ottawaense* OO99^{T}; 6, *B. amphicarpaeae* 39S1MB^{T}; 7, ***B. symbiodeficiens*** **85S1MB^{T}**; 8, ***B. symbiodeficiens*** **101S1MB**; 9, ***B. symbiodeficiens*** **65S1MB**; M1, M2 and M3 represent 1kb, 100bp and 50bp marker ladders (FroggaBio, Ontario, Canada), respectively.

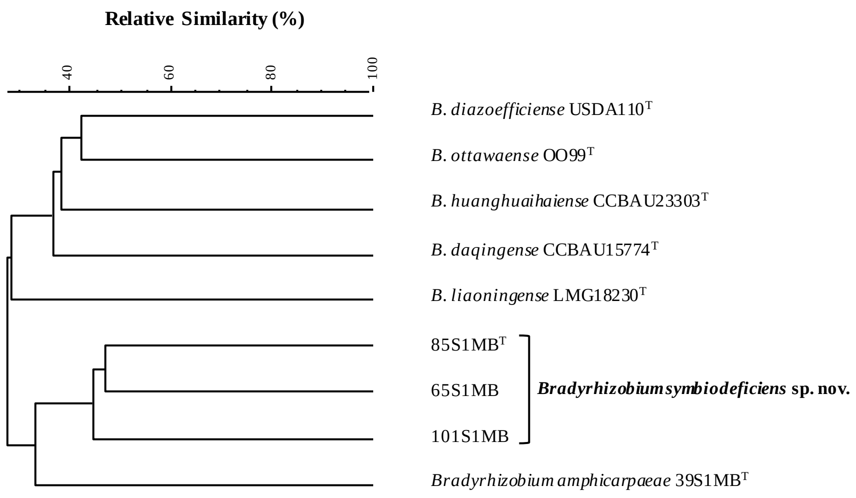

Supplementary Fig. S9 Dendrogram (constructed using UPGMA and the Dice coefficient) of representative strains of *Bradyrhizobium symbiodeficiens* and reference taxa based on the combined character matrix of RAPD fingerprints generated by four random primers (P1, P2, P3 and P5).

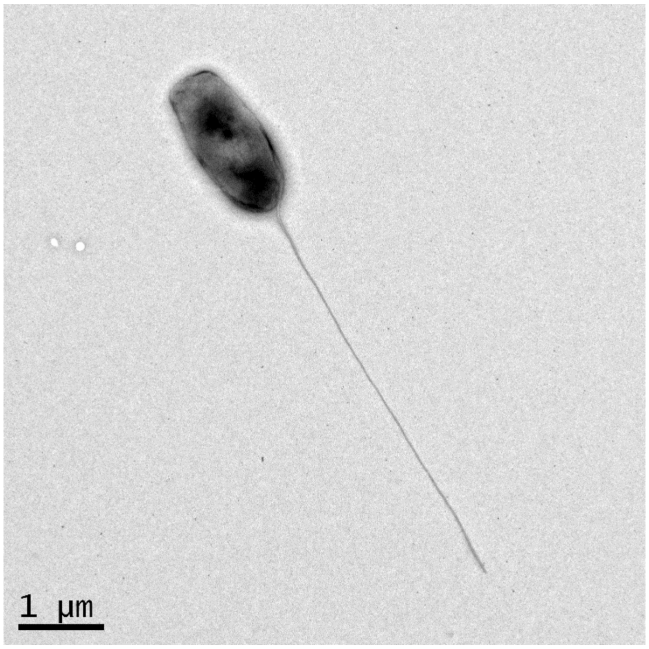

Supplementary Fig. S10. Transmission electron micrograph showing a cell of *B. symbiodeficiens* sp. nov., 85S1MB^{T} with sub-polar flagellum.

Average cell size, 1.75 X 0.83 µm (based on 10 measurements).
